# Early-induced diabetic obese rat MACAPOS 2

**DOI:** 10.1186/s12902-022-01252-8

**Published:** 2023-03-20

**Authors:** Joseph Ngakou Mukam, Clémence Mvongo, Sandrine Nkoubat, Gaëtan Olivier Fankem, Adamou Mfopa, Paul Aimé Noubissi, Michel Archange Fokam Tagne, René Kamgang, Jean-Louis Essame Oyono

**Affiliations:** 1 Animal Physiology Laboratory, Faculty of Science, University of Yaoundé I, Yaoundé, Cameroon; 2 Department of Life Sciences, High Teacher Training College of Bertoua, University of Bertoua, Bertoua, Cameroon; 3 Laboratory of Human Metabolism and non-Communicable Diseases, Institute of Medical Research and Medicinal Plants Studies (IMPM), Yaoundé, Cameroon; 4 Department of Animal Biology and Conservation, Faculty of Science, University of Buea, Buea, Cameroon; 5 Department of Biological Science, Faculty of Science, University of Ngaoundéré, Ngaoundéré, Cameroon

**Keywords:** Diabetes, High Fat Diet, Streptozotocin, Obese rat, MACAPOS 2

## Abstract

**Background:**

Diabetes mellitus is a metabolic disease characterized by an abnormally high blood glucose level. Glucose intolerance and insulin resistance are two characteristics that promote the onset and development of type 2 diabetes. The aim of this study was to create a diabetic rat model from obese rat MACAPOS 2.

**Methods:**

A group of rats was subjected to a high-fat diet (HFD) compared to a control group (NC) which received a normal diet. After 16 weeks of HFD, Lee index was calculated, obese rats were subjected to an oral glucose tolerance test (OGTT) and insulin tolerance test (ITT). One group of HFD rats (HFDZ) received streptozotocin 22.5 mg/kg (iv). One week later, weight gain, water and food intakes, urine volume and fasting blood glucose levels were evaluated. Animals were also subjected to glucose tolerance and insulin tolerance tests.

**Results:**

After 16 weeks of HFD, rats became obese, glucose intolerant and resistant to insulin. The body weight of rats was significantly high (+ 26.23%) compared to normal rats, glycemia remained significantly high (+ 45.46%, *P* < 0.01) two hours after administration of glucose in high-fat diet rats, water intake and urine volume were comparable to those of NC. In HFD, the streptozotocin injected after one week (HFDZ), amplified glucose intolerance. During ITT, glycemia remained significantly (*P* < 0.01) high from 15 min; and did not vary during the 60 min of ITT. The fasting glycemia one week after streptozotocin injection was significantly high (288 mg/dL) compared to HFD (114 mg/dL), associated whit a significant (*P* < 0.01) increase in water intake and 24 h urine volume.

**Conclusion:**

These results showed that MACAPOS 2 associated with a low dose of streptozotocin (22.5 mg/dL) early leads to the diabetes in obese albinos *Wistar* rats and could be a real model to study the type 2 diabetes mellitus.

## Background

Diabetes mellitus is one of the most common metabolic diseases that is caused by an absolute or relative deficiency in insulin secretion and/or insulin action [1, 2]. In other to study, understand and manage this metabolic affection, many diabetic animal models are used by the scientific community to evaluate the efficiency of many potential substances in diabetes management. Among the many existing models, the type 1 model is obtained by injection of chemical substances (Streptozotocin, alloxan) that lead either to a total or partial destruction of the pancreatic beta-cells [3–5]. Streptozotocin (STZ) is used at high doses to induce diabetes in rodents (animal models of insulin-dependent diabetes mellitus) characterized by high fasting blood glucose levels. Type 2 diabetes (T2D) is caused by a combination of genetic factors related to insulin resistance and environmental factors such as obesity, lack of exercise, and stress [6]. Different T2D animal models remain indispensable for discovering, validating, and optimizing novel therapeutics for their safe use in human diabetes management. Many genetic and spontaneous models of type 2 diabetes are developed; however, these models are not available in developing countries and, many of these models do not really reflect the human cases of diabetes. Nongenetic models are more like human T2D disease with regard to the initiation and progression phases. The high-fat diet is known to induce obesity, insulin resistance and glucose intolerance in albinos *Wistar* rats [7]. The MACAPOS 1 diabetic and MACAPOS 2 obese models’ rats were created using 16 weeks of Cameroon’s local hypercaloric diet [8]. MACAPOS 1 combined with dexamethasone reduced to 10 weeks the induction period and induced more severe hyperglycemia [9]. Unlike MACAPOS 1, MACAPOS 2 leads to common obesity: after 16 weeks of high-fat diet, the animal became obese, glucose intolerant and insulin resistant. MACAPOS 2 rats do not develop real fasting hyperglycemia [7, 8]. Streptozotocin, at high doses, causes beta islet cell death by acute oxidative stress [5, 10]. However, the injection of a low dose in rats previously subjected to a high-fat diet leads to the onset of type 2 diabetes [11, 12].

In the aim of finding a new animal model of diabetes study that is closer to the human model in our local context, the present study was undertaken to create a combined model MACAPOS 2-streptozotocin.

## Methods

### Animals

*Wistar* rats were bred in the animal house of the Laboratory of Human Metabolism and non-Communicable Diseases. They were maintained under natural light /dark cycle and fed with a standard local diet (3.400 kcal: carbohydrates 50–55%, fats 15–20%, proteins 25–30%), and had access to water ad libitum [8]. In vivo experiments were conducted with the approval from the institutional committee of the Cameroonian Ministry of Scientific Research and Innovation which has adopted the guidelines and regulations of the European Union on Animal Care (CEE Council 86/609) [13]. The ARRIVE guidelines were also observed.

### MACAPOS 2 obese rats

Six weeks old male albinos *Wistar* rats were selected for obesity induction. They were randomly divided into two groups, normal control (NC, submitted to the standard diet) and high-fat diet (HFD) group (Table 1), with free access to water [8]. After four (4) months under the respective diets, Lee index and body weight gain allowed us to select obese rats [7]. They were then subjected to oral glucose and insulin tolerance tests to select glucose intolerant and insulin resistant ones.

**Table 1:** Diet compositions and their caloric intake (energy)

Groups	Normal Diet (ND)	High Fat Diet (HFD)
Maize	250	80
Wheat	400	110
Soya bean	150	280
Steeped cassava	–	220
Fish flour	100	30
Sucrose	–	50
Palm oil	–	200
Bones flour	10	20
Cabbage palm cake	80	–
Vitamins complex	10	10
Energy (Kcal/Kg)	3400	4730

The components were obtained from Yaoundé (Cameroon) local market and are expressed in g/kg of diet

Lee index (Li) was calculated, using the body weight (bw) and Naso-anal length (Lna) as follow [9–14]:

$$\textrm{Li}=\frac{\sqrt[3]{bw}}{Lna}$$

### Obese diabetic rats

Obese rats received a unique dose of streptozotocin (22.5 mg/kg *i.v*. Sigma Aldrich). To ensure the implication of the diet on the genesis of diabetes, another group consisting only of normal rats was used in comparison to the obese rats’ group; and these normal rats also received streptozotocin. One week after streptozotocin administration, weight gain, water and food intakes, 24 hours urine volume and fasting blood glucose levels were recorded. All animals were subjected to glucose and insulin tolerance tests.

### Oral glucose tolerance test (OGTT)

After 12 h fasting the rats received per os 2.5 mg/kg of glucose. Prior to glucose administration, blood glucose was estimated, and then at 30, 60, and 120 min after oral glucose administration [8]. Glycemia was evaluated using test strips ACCU-Chek Active and a glucometer of the same brand.

### Insulin tolerance test (ITT)

After a 12 h fasting period, the blood glucose level of each animal was evaluated; each animal received 2 UI/kg bw S.c insulin (Insulin Actrapid Human HM). The glycemia was then estimated at 10, 20, 30, and 60 min after insulin administration [9].

### Statistical analysis

The results were expressed as mean ± standard error of the mean. The statistical analyses were performed by one-way analysis of variance (ANOVA) associated with the Turkey test followed by the Dunnett test, using the computer Graphpad Prism 8.0.1. The difference between and within various groups was significant with *P* < 0.05.

## Results

### Body weight, water and food intakes, urine volume

**Fig. 1:**
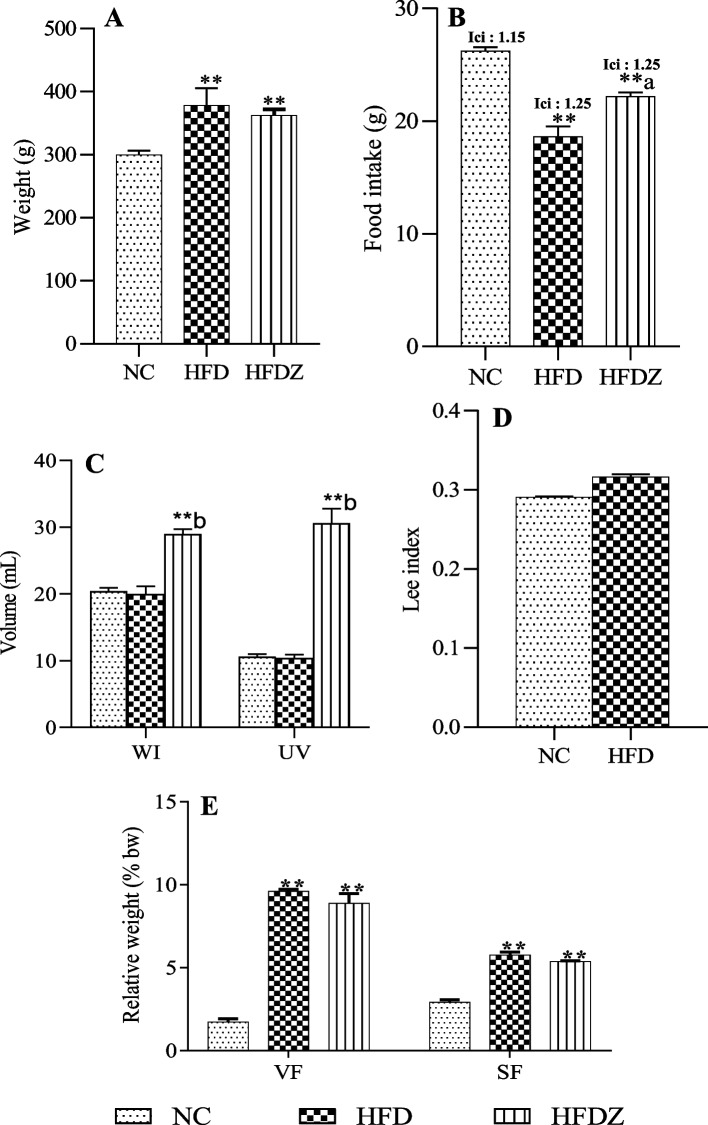
Body weight (**A**), food intake (**B**), water intake (WI) and 24 h urine volume (UV) (**C**), Lee index (**D**), visceral (VF) and subcutaneous (SF) fats (**E**) of 16 weeks high-fat diet (HFD), and high-fat diet plus streptozotocin (HFDZ). Ici: initial caloric intake

After 16 weeks of a high-fat diet, rat body weight significantly increased compared to those submitted to a normal diet (NC): (+ 26,23%, *P* < 0.01, Fig. 1A). The Streptozotocin administration caused a non-significant reduction in the weight of rats submitted to high-fat diet (Fig. 1A). In HFD, food intake was significantly low (*P* < 0.01) compared to NC. HFDZ rats’ food intake significantly increased compared to HFD, but remained significantly low compared to NC (Fig. 1B). The initial caloric intake was higher in HFD (1.25 kcal/g bw) compared to NC (1.15 kcal/g bw) (Fig. 1B). Water intake and 24 h urine volume of HFD were not significantly different to NC (Fig. 1C) but were significantly (*P* < 0.01) increased in HFDZ rats compared to NC and HFD rats (Fig. 1C). In HFD rats, the Lee index was greater than 0.3, and less than 0.3 in NC (Fig. 1D). The relative weights of visceral and subcutaneous fat were remarkably high in animals subjected to a high-fat diet compared to NC. Also, streptozotocin administration brought a significant increase of fat relative weight in HFDZ animals (Fig. 1E).

### Obese rat MACAPOS 2

**Fig. 2:**
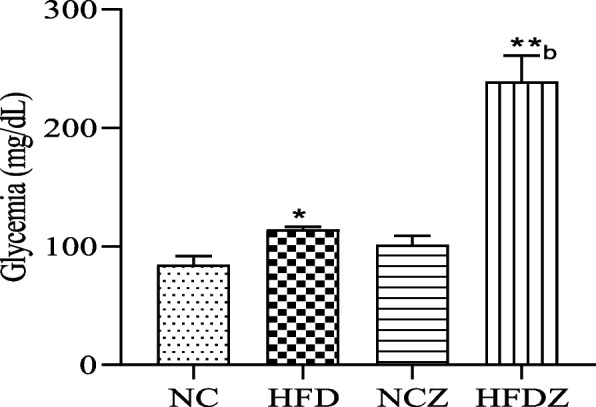
Fasting glycemia of 16 weeks high-fat diet (HFD) and high-fat diet plus streptozotocin (HFDZ). NC: normal control rats; NCZ: normal rats receiving streptozotocin. Significant difference: **P < 0.01, *P < 0.05 compared to NC; ^b^*P* < 0.01 compared to HFD

**Fig. 3:**
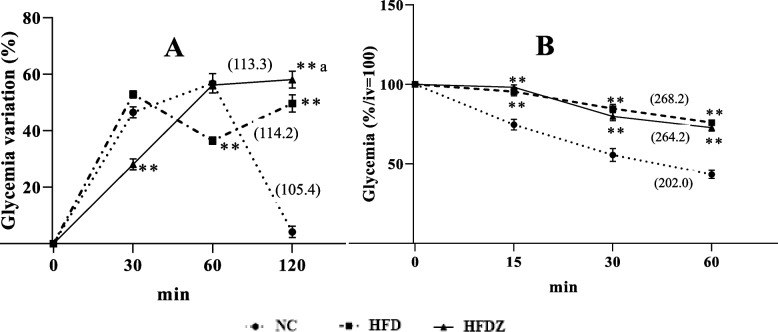
Glycemia of oral glucose (**A**) and insulin tolerance tests (**B**) of 16 weeks high-fat diet (HFD) and high-fat diet plus streptozotocin (HFDZ), expressed in percentage of variation for A, and in percentage of the initial value (iv = 100%) for B. NC: normal control rats. Significant difference: **P < 0.01 compared to NC; ^a^*P* < 0.01, ^b^*P* < 0.01 compared to HFD. (): Area Under the Curve

High-fat diet fed rats presented high blood glucose level (110.0 ± 1.6 mg/dL) compared to normal diet fed control group (84.0 ± 3.0 mg/dL, Fig. 2). Glycemia variation remained significantly high (+ 45.46%, *P* < 0.01) 2 h after administration of glucose in high-fat diet rats compared to the NC rats (Fig. 3A). After insulin administration, glycemia remained significantly (*P* < 0.01) high from 15 min in high fat diet rats compared to those of NC (Fig. 3B).

### Diabetic rat MACAPOS 2

Streptozotocin administration at 22.5 mg/kg (iv) did not significantly increase blood glucose level in normal rats but increased glycemia in HFD rat from 110 mg/kg to 288 mg/dL, (*P* < 0.01, Fig. 2). Streptozotocin amplified glucose intolerance; blood glucose levels remained significantly high at 60 min (+ 20.3%, *P* < 0.01) and 120 min (+ 8.5%, *P* < 0.05) in HFDZ rats compared to those of HFD (Fig. 3A). In HFDZ rats as HFD rats, blood glucose levels remained high during the 60 min of ITT (Fig. 3B).

## Discussion

This study was undertaken to set up in our local context, a new diabetes model close to the human model. Submitted to a high-fat diet, animals became obese after 16 weeks. A high-fat diet leads to obesity, hyperinsulinemia, and altered glucose homeostasis which may be due to insufficient compensation by the beta cells of the pancreatic islets [15]. The weight gain and increase in Lee index observed in HFD rats are generally due to an increase in fat mass, which could also justify the glucose intolerance and insulin resistance of these rats. In the high-fat diet, the total energy intake was greater compared to the normal diet. This could explain why despite the low food intake, the HFD animals gained more weight. The increase of the body fat tissue may not only result from the fat content of the diet, but also from the energy intake, which might lead to various metabolic alterations such as insulin resistance, reduction of lipolytic activity in fat tissue, and impairment of mitochondrial metabolism [16]. Metabolomic studies on HFD-fed mice have shown incomplete oxidation of fatty acids, accompanied by an increase in whole-body fatty acid oxidation [16, 17]; HFD led to the accumulation of fatty acid oxidation byproducts in skeletal muscle, in turn, this contributes to insulin resistance in muscle [16]. In fact, the energy used by the muscle is produced primarily by free fatty acids oxidation with muscle glycogen stores remaining intact, and repression of glycogen synthase [18]. These mechanisms contribute to a rise in blood glucose levels by reducing the muscle capacity to use and store glucose and an increase in hepatic glucose release [19]. Furthermore, in obesity, oxidative stress, inflammation, and excessive production of certain adipokines such as resistin, increase insulin resistance [20–22]. Dietary nutrients (sucrose and palm oil) found in the administered diet might have a profound influence on insulin action, also, it could be associated with impaired mitochondrial function; with the production of malonyl-CoA, which reduces GLUT4 efficiency [23]. These mechanisms might contribute to the development of the observed glucose intolerance [8]. MACAPOS 2 is known to induce dyslipidemia, glucose intolerance and insulin resistance [7, 8, 24].

Administration of a low dose of streptozotocin (22.5 mg/kg) did not induce diabetes in normal rats, but caused in HFD rats, within a week, a diabetes state characterized by fasting hyperglycemia. Streptozotocin at a high dose (40 mg/kg) induces hyperglycemia by a selective toxicity activity on pancreatic beta cells [5, 25, 26]. Streptozotocin damages the pancreatic beta cells and induces hyperglycemia; this effect is not observed at low doses [5]. In this study, a low dose of streptozotocin (22.5 mg/kg) developed fasting hyperglycemia in obese rat MACAPOS 2. This result could be due to diet-induced glucose intolerance and insulin resistance which are characteristic of a common obesity in human. This common obesity created a favorable stage for the onset of hyperglycemia and type 2 diabetes [27]. Generally, STZ-induced type 1 diabetes is also characterized by body mass loss [28]; in our studied model, the body mass, visceral and subcutaneous fat remained significantly higher after STZ administration. This is also the case in the type 2 diabetes model. The hyperglycemia observed in MACAPOS 2 rat was accompanied by diabetes characteristics such as the increase in water and food intake and 24 h urine volume. Hyperglycemia causes an increase in urine volume due to osmotic diuresis [29], which also explains the increase in water intake. In fact, polyuria leads to dehydration, which causes a strong feeling of thirst [29]. These last symptoms associated with insulin resistance and glucose intolerance, confirm a real type 2 diabetes as observed in human beings.

In conclusion, the Cameroon’s local diet associated with a low dose of streptozotocin quickly leads in obese MACAPOS 2 albinos *Wistar* rats to type 2 diabetes characterized by fasting hyperglycemia, glucose intolerance, insulin resistance, as well as an increase in urine volume and water intake. This model could be used as a model of type 2 diabetes mellitus in scientific studies.

## Data Availability

All data generated or analyzed during this study are included in this manuscript. These data are available from the corresponding author upon request.

## Abbreviations

MACAPOS: Maize, Cassava, Palm Oil, and Sugar
HFD: High Fat Diet
HFDZ: High Fat Diet plus streptozotocin
STZ: Streptozotocin
NC: Normal Control
ITT: Insulin Tolerance Test
OGTT: Oral Glucose Tolerance Test
T2D: Type 2 Diabetes
